# Involving private healthcare practitioners in an urban NCD sentinel surveillance system: lessons learned from Pune, India

**DOI:** 10.3402/gha.v9.32635

**Published:** 2016-10-18

**Authors:** Mareike Kroll, Revati Phalkey, Sayani Dutta, Sharvari Shukla, Carsten Butsch, Erach Bharucha, Frauke Kraas

**Affiliations:** 1 Institute of Geography, University of Cologne, Cologne, Germany; 2 Division of Epidemiology and Public Health, University of Nottingham, Nottingham, United Kingdom; 3 Institute of Environment Education and Research, Bharati Vidyapeeth University, Pune, India; 4 Symbiosis Institute of Health Sciences, Symbiosis International Institute, Pune, India

**Keywords:** sentinel surveillance system, non-communicable disease, private healthcare sector, low- and middle-income countries, research to policy

## Abstract

**Background:**

Despite the rising impact of non-communicable diseases (NCDs) on public health in India, lack of quality data and routine surveillance hampers the planning process for NCD prevention and control. Current surveillance programs focus largely on communicable diseases and do not adequately include the private healthcare sector as a major source of care in cities.

**Objective:**

The objective of the study was to conceptualize, implement, and evaluate a prototype for an urban NCD sentinel surveillance system among private healthcare practitioners providing primary care in Pune, India.

**Design:**

We mapped all private healthcare providers in three selected areas of the city, conducted a knowledge, attitude, and practice survey with regard to surveillance among 258 consenting practitioners, and assessed their willingness to participate in a routine NCD surveillance system. In total, 127 practitioners agreed and were included in a 6-month surveillance study. Data on first-time diagnoses of 10 selected NCDs alongside basic demographic and socioeconomic patient information were collected onsite on a monthly basis using a paper-based register. Descriptive and regression analyses were performed.

**Results:**

In total, 1,532 incident cases were recorded that mainly included hypertension (*n*=622, 41%) and diabetes (*n*=460, 30%). Dropout rate was 10% (*n*=13). The monthly reporting consistency was quite constant, with the majority (*n*=63, 50%) submitting 1–10 cases in 6 months. Average number of submitted cases was highest among allopathic practitioners (17.4). A majority of the participants (*n*=104, 91%) agreed that the surveillance design could be scaled up to cover the entire city.

**Conclusions:**

The study indicates that private primary healthcare providers (allopathic and alternate medicine practitioners) play an important role in the diagnosis and treatment of NCDs and can be involved in NCD surveillance, if certain barriers are addressed. Main barriers observed were lack of regulation of the private sector, cross-practices among different systems of medicine, limited clinic infrastructure, and knowledge gaps about disease surveillance. We suggest a voluntary augmented sentinel NCD surveillance system including public and private healthcare facilities at all levels of care.

## Introduction

The increasing burden of non-communicable diseases (NCDs) is one of the most pressing global public health challenges (1). In India, NCDs contributed to an estimated 61% of all deaths in 2014 (2), with projections indicating a further rise to 67% in 2030 (3). The four leading causes of death globally (2) and in India (4) in descending order are cardiovascular diseases, chronic respiratory diseases, cancers, and diabetes. The rising morbidity and mortality through NCDs pose a big challenge in India. First, NCDs impact people at younger ages compared to high-income countries, increasing the healthy life years lost (4). Second, NCDs have a large socioeconomic impact due to long-term treatment costs and loss of productivity (5). Third, NCDs – especially if inadequately controlled – increase the risk of comorbidities and hence jeopardize the control of communicable disease. For example, about 15% of all tuberculosis cases in India are attributable to diabetes (6).

Despite the increasing impact of NCDs on public health in India, lack of comprehensive quality data hampers – as in many other low- and middle-income countries (7) – the planning process for NCD prevention and control (8). So far, the majority of national surveillance programs focus on communicable diseases (9). Health programs with a focus on NCDs have weak surveillance components. The National Programme for Prevention and Control of Cancer, Diabetes, Cardiovascular Diseases and Stroke, launched in 2010, focuses on the prevention and control of NCDs, but includes a weak surveillance component (10). Although the Integrated Disease Surveillance Program has a component on NCDs, only one NCD risk factor survey has been conducted so far (2007) in 7 of the 29 states and 7 Union Territories in the country (11, 12). The National Cancer Registry Program (2013) includes 28 population-based cancer registries and seven hospital-based registries (13). In addition, information is irregularly collected through population-based surveys such as the National Family Health Survey (14), with data collection on few NCDs (diabetes, asthma, thyroid disorders) and risk factors (nutrition, tobacco, and alcohol consumption).

Another drawback for disease surveillance efforts in India is the excessive focus on the public sector despite the private sector's dominance in healthcare provision (15). Private practitioners are the preferred first contact point and provide nearly 80% of outpatient and 60% of inpatient care in India (16). The private sector is heterogeneous and covers various formal systems of medicine such as allopathy, homeopathy, ayurveda, and unani (17). Challenges exist in the regulation, quality, accountability, and cooperation between the different systems of medicine and their collaboration with the government sector (15, 18). The integration of the private sector in routine disease surveillance is urgently needed to increase the data range. Such a system should also capture differences in health status and access to healthcare of different socioeconomic groups, especially in urban areas (19, 20).

Against this background, the objective of the study was to conceptualize a prototype for an urban NCD sentinel surveillance system; to test it in three preselected areas in Pune, India; and to evaluate its implementation. The system was designed in a way that it did not duplicate existing programs, but rather supplemented them. Private practitioners providing primary care were identified as sentinel sites, and data on incident cases were collected over 6 months (March–September 2014). We present the main observations made during the process of implementing the system and report the lessons learned that should be considered in designing routine urban NCD surveillance systems in the future.

## Methods

### Surveillance approach and study design

Based on a literature review on NCD surveillance (7) and on discussions with key informants in Pune, a design for a sentinel surveillance system to capture first-time diagnosed NCD cases was developed. Private practitioners providing primary care (general practitioners, general physicians, and pediatricians of different systems of medicine [allopathy, ayurveda, and homeopathy]) holding graduate and postgraduate degrees were included as sentinel reporting units (RUs). The focus was on primary care because these practitioners often serve as the first point of care (15).

Ten NCDs were selected based on the major causes of death in India (4) and recommendations of the WHO (21): cardiovascular disease (hypertension, cerebrovascular diseases, and ischemic heart disease), chronic respiratory disease (asthma and chronic obstructive pulmonary disease [COPD]), cancer (breast, cervical, lung, and oral), and diabetes. Practitioners were requested to record only those patients with a first-time diagnosis of any of the selected diseases to avoid duplication of cases if patients visited additional practitioners for care.

Socioeconomic status is an important determinant of NCDs and also affects the access to NCD care. Because practitioners were hesitant to record income and occupation of the patients (22), we used educational qualification as the proxy indicator for the socioeconomic status of the patient.

### Sampling framework

**Fig. 1:**
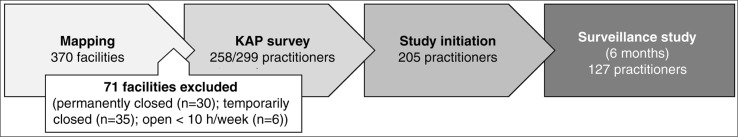
Selection process of reporting units for the surveillance study.

The sampling process was conducted in four stages (Fig. 1). 1) Due to lack of a common registration platform for private practitioners in Pune, all private healthcare facilities (full sampling) in three identified areas were mapped using Mobile Mapper 6W/GIS (*n*=370). 2) A knowledge, attitude, and practice (KAP) survey (full sampling) with respect to disease surveillance was conducted in July–August 2013 among 258 practitioners (86% of all running facilities), and their interest to participate in the proposed surveillance study was recorded (22). 3) In total, 205 practitioners (79% of all KAP study participants) stated an interest and were visited in March 2014 with a prior appointment to reconfirm their interest and to introduce the surveillance study. In facilities with more than one practitioner, only the KAP interviewee was asked to participate in the surveillance study. 4) In total, 127 practitioners (49% of all KAP study participants) gave their final consent to participate in the 6-month surveillance study.

### Data collection process

During the KAP survey, a majority of the respondents (*n*=170 of 201 respondents, 85%) stated their preference for a paper-based reporting format. In line with this preference, a paper-based register with a unique ID containing 100 reporting forms was provided to each RU. Each form contained eight items: date of visit, age, gender, residential area (on subward level), level of education, diagnosis for selected 10 diseases, diagnosis (presumptive or confirmed), and treatment (initial treatment at RU or referral at first instance). We did not collect any patient-identifiable information. All data were anonymized at the point of collection. The register design was pretested for 1 week.

The process, duration, and register design were clearly explained to all participants at the first visit, and a written consent was obtained. Printed guidelines on how to fill the register and contact numbers of the investigators were also provided. Data were collected physically on site by four researchers monthly using a standard protocol. During the first and the sixth data collection, standardized feedback about the register and study design were collected from each RU. Data were entered into a database each month after each collection round, and the complete database was checked for entry errors by two other investigators. Computer-based checks were used to clean the data, and inconsistencies were resolved on the basis of information recorded.

### Data processing and evaluation of the study

Descriptive analyses were done using Microsoft^®^ Excel 2011 to assess 1) the characteristics and participation of the RUs, 2) the process of setting up and running the surveillance system, and 3) the reported data. Regression analyses were carried out using STATA version 7.0 to study the univariate associations of practitioners' characteristics and available infrastructure with practitioners' participation in the surveillance study, adjusting for years of experience, system of medicine, and other factors. Logistic regression models were used to assess whether associations were independent of potential confounding factors.

## Results

### Participation in surveillance study and characteristics of RUs

Of the 258 KAP survey participants, 127 practitioners (49%) gave written consent for participation in the surveillance study. The participation rate was 52% (*n*=44), 51% (*n*=58), and 42% (*n*=25) among homeopathic, ayurvedic, and allopathic practitioners, respectively. The majority of the enrolled practitioners (*n*=98, 77%) had a graduate degree and were trained in ayurvedic medicine (*n*=58, 46%) (Table 1). Clinics were predominantly small, with a low number of staff and only very basic infrastructure (Table 1). Only a few practitioners used electronic medical records (*n*=15, 12%).

**Table 1:** Features of participating practitioners and their clinics (*n*=127) (*n* (%))

Features of participating practitioners, *n* (%)			Features of clinics, *n* (%)		
System of medicine	Allopathy	25 (20)	No. of practitioners	≥2 practitioners	41 (32)
	Ayurveda	58 (46)			
	Homeopathy	44 (35)	Inpatient treatment	No. of clinics	25 (20)
Qualification	Graduate	98 (77)		Mean no of beds	6.9 (*n*=25)
	Postgraduate	29 (23)	Clinic staff	Receptionist	54 (43)
No. of patients	Mean/day	27.4		Paramedic staff	27 (21)
Medical records	Always	93 (73)	Infrastructure	Computer	48 (38)
	Sometimes	26 (21)		Generator	60 (47)
Type of records	Electronic	15 (12)		ECG	31 (24)
	Paper	104 (82)		USG	3 (2)
Medical record	Gender, age, diagnosis	61 (48)		X-ray	11 (9)

Logistic regression showed that practitioners who always maintained patients’ records were more likely to participate in the surveillance study (odds ratio, 2.287; 95% confidence interval [CI], 1.162–4.498) compared to those who did not maintain records regularly. However, years of experience, education of practitioners, system of medicine, location, infrastructure (phone, electricity, computer and generator backup), and availability of human resources (paramedic staff and receptionist) were no significant determinants in the analysis. The results remained similar when we adjusted the analysis separately for gender, years of experience, age of the practitioners, and location.

### Evaluation of the participation process

#### Process monitoring

Consistency in participation

During the 6-month surveillance study, 13 practitioners (10%) discontinued their participation, with the majority of them discontinuing in round five (*n*=6, 46%) (Table 2). The highest dropout rate was observed among allopathic practitioners (*n*=5, 20%). The main reasons stated were lack of time (*n*=3, 23%), frequent absence (*n*=3, 23%), lack of first-time diagnosed cases (*n*=2, 15%), and relocation or closure of the clinic (*n*=2, 15%).

**Table 2:** Average and total number of reported case/collection round per RU according to system of medicine

	Allopathy, *n* (%)	Ayurveda, *n* (%)	Homeopathy, *n* (%)	Total, *n* (%)
No. of RUs	25	58	44	127
No. of dropouts	5 (20)	5 (9)	3 (7)	13 (10)
No. of cases	388 (30)	582 (45)	313 (25)	1283 (100)
Ø cases/RU	17.4	10.2	7.4	10.6
Avg. no. of submitting RUs (per round)	12.2 (49)	23.7 (41)	15.8 (36)	51.7 (41)
No. of cases per RU (6 months)				
0 cases	6 (24)*^4^	14 (24)*^4^	8 (18)*^2^	28 (22)
1–10 cases	9 (36)	24 (41)*^1^	30 (68)*^1^	63 (50)
11–20 cases	4 (16)	12 (21)	4 (9)	20 (16)
21–30 cases	4 (16)*^1^	5 (9)	1 (2)	10 (8)
>30 cases	2 (8)	3 (5)	1 (2)	6 (5)

RU =reporting unit.

* Indicates no. of dropouts.

Cooperation

The accessibility of the RUs was measured through the number of phone calls (average of 1.5 calls/practitioner per round), visits (1.3 visits/practitioner per round) and the waiting time in the RU (8 min/practitioner per round). No linear changes emerged for all three issues that might indicate a decreasing or increasing cooperation over the 6-month period. Cooperation was also not related to system of medicine or qualification. Some practitioners were more difficult to access because of lack of time or non-cooperation. For example, 76 RUs (60%) were always just visited once per round; others had to be visited more often. The waiting time also varied between the RUs ranging from 0 to 60 min and was the highest for allopathic practitioners (11 min).

Reporting consistency

On average, 52 RUs (41%) submitted cases during each round. The monthly reporting consistency was quite constant and ranged from 47 (37%) to 56 (44%) RUs submitting cases. The absolute number of cases submitted per RU during the 6 months ranged from 0 cases (*n*=28, 22%) to a maximum of 119 cases submitted by an allopathic physician, followed by a homeopathic practitioner with 87 cases. The majority of practitioners submitted 1–10 cases (*n*=63, 50%) (Table 2). Of 36 (28%) practitioners who submitted a more**-**than**-**average number of cases, 10 (28%) were allopath, 20 (56%) were ayurvedic, and 6 (17%) were homeopathic practitioners.

The average number of reported cases over the 6-month period was 10.6 per practitioner (Table 2). Case reporting remained constant over the reporting period, ranging from 1.3 to 2.6 cases per round. Although the highest number of cases was the submitted by ayurvedic practitioners (*n*=582, 45%), the average number of cases was the highest among allopathic practitioners (17.4 cases/RU).

The total number of first-time diagnosed NCD patients seen by a practitioner was related to work experience and patient volume, factors that are also mutually dependent. Practitioners with 20–29 years of experience and more than 1,500 patients per month submitted the maximum number of cases (48 cases/RU).

To identify reasons for the deviating number of cases, practitioners with less than five cases during the first five collection rounds (*n*=58, 46%) were asked during the final evaluation to provide an explanation. Ayurvedic and homeopathic practitioners (*n*=25, 43%) mainly said that they would rarely see new NCD cases, either because they practiced pure alternative medicine (only providing additional treatment for NCD patients) or mainly diagnosed patients with communicable diseases. One allopathic physician explained he would mainly see prediagnosed patients from general practitioners. Other practitioners (*n*=5, 9%) named reasons as such low number of patients, irregular or limited opening hours, or temporary closure of the clinic. Seventeen practitioners (29%) said they had not entered all cases due to negligence (*n*=14) or lack of time (*n*=3).

Data quality

Of all forms, at least one value was missing in 249 forms (19%). Of these forms, 36 (3% of all forms) had more than one value missing. The most frequent value missing was education (*n*=70, 7%) (Table 3). Considering only the essential variables for surveillance (age, date, gender, diagnosis), 9% (*n*=110) of all forms had missing values. The month**-**wise analysis of missing values does not show a linear trend: the number of missing values was the lowest during round 1 (13%) and 6 (16%) and varied between 23 and 24% in round 2–5.

**Table 3:** Data completeness: number of values missing (*n*=1,283)

System of medicine	*n* (%)
Education	90 (7)
Age	76 (6)
Residential area	30 (2)
Confirmation of diagnosis	26 (2)
Referral of patient	22 (2)
Date (missing/incomplete)	21 (2)
Gender	21 (2)
Diagnosis	2 (0)

Some practitioners made notes on the form, e.g. in case of an unusual diagnosis (such as breast cancer in a male patient, hypertension in a very young patient) to show that this diagnosis was not a data entry error, or specified laboratory tests to demonstrate accuracy of the diagnosis. This shows the high willingness of some practitioners to contribute to NCD surveillance with valid and complete data.

The practitioners were advised to enter each case during or directly after the patient visit to ask the patient for details such as educational degree. According to own observations, the forms were ready for collection in 45% of all RUs (*n*=57), among allopathic practitioners even in 56% of all RUs (*n*=14). About 38% of the practitioners (*n*=43) used their own records to enter cases, thereby increasing the risk of wrong data because their medical records often did not cover all required information (Table 1). About 10% of the practitioners (*n*=11) said during the final evaluation that they had filled the register later based on memory, mainly when the data was about to be collected by the field staff. This increases the risk of wrong entry and underreporting.

During the final evaluation, practitioners were asked whether they had entered all relevant cases over the past 6 months. Complete recording was claimed by 28% of the practitioners (*n*=32), whereas 36% (*n*=41) said that they entered less than 75% of all cases (Table 4). Fifteen percent of ayurvedic (*n*=8) and homeopathic (*n*=6) practitioners entered even less than 50% of all cases. The main reasons for not entering all cases were lack of time/patient load (*n*=39, 48%) and forgetfulness to enter some cases (*n*=27, 33%).

**Table 4:** Self-evaluation by the RUs after month 6: percentage of all recorded cases from all relevant cases seen during the surveillance study

	Allopathy, *n* (%)	Ayurveda, *n* (%)	Homeopathy, *n* (%)	Total, *n* (%)
All cases	3 (15)	18 (34)	11 (27)	32 (28)
75–99%	11 (55)	15 (28)	15 (37)	41 (36)
50–74%	6 (30)	12 (23)	9 (22)	27 (24)
<50%	0	8 (15)	6 (15)	14 (12)
Total	20 (100)	53 (100)	41 (100)	114 (100)

RU=reporting unit.

#### Participants’ evaluation of the surveillance study

The practitioners judged the register design as easy to navigate and time-efficient to fill in. Practitioners suggested to add the following information to the form: additional medical information (risk factors, medical history, comorbidities, follow-ups, symptoms, medication, compliance) (*n*=27, 24%); other diseases (*n*=23, 20%); patient information (exact address, unique ID, occupation) (*n*=15, 13%); and further detailed information such as type of disease, prediagnosed cases/secondary diagnosis, specification of test/laboratory report, separate formats for pediatric, and adult and geriatric patients (*n*=12, 11%). Regarding the selection of RUs, some graduate practitioners suggested to only involve postgraduates and specialists or tertiary hospitals in surveillance because many patients with symptoms for NCDs would directly go to a specialist. In contrast, some postgraduate practitioners said that they would mainly see prediagnosed patients referred from graduate general practitioners. This indicates that there is no direct linkage between qualification of practitioner and diagnosis of NCD cases. Suggestions regarding the data collection mainly addressed the need for regular contact and interaction, but they also addressed training and awareness building.

At the end of the study, 91% (*n*=104) of the practitioners thought that the surveillance system design can be transferred to the city. Similarly, 93% of the practitioners (*n*=106) said they would be willing to participate in a routine NCD surveillance system, based on their experience in the surveillance study. Among allopathic practitioners, agreement was 100% (*n*=20). However, concerns were raised that the success of the system would depend on the willingness of the practitioners, the time required, and the support they would get. They requested a transparent system with good access to the responsible institution.

A majority of the practitioners (*n*=70, 64%) who showed an interest to participate in a routine surveillance system would be willing to send data electronically, either through computer or smartphone-based application; 64 of them (91%) would also install and use a standardized format to submit data electronically. Others (*n*=39, 36%) preferred paper-based systems. No significant variation according to system of medicine was observed in this respect. Only 31% (*n*=34) of the respondents said that financial, material, or non-material incentives should be provided for active participation in regular surveillance. A majority of the practitioners (*n*=82, 75%) said that Continued Medical Education points for participating in surveillance might be a good incentive.

### Reporting outcome: number of cases, treatment, and referral patterns

In total, 1,532 first-time diagnoses of the selected 10 conditions were reported in 1,283 individuals, including concurrent diagnoses of multiple conditions in the same patient (*n*=224, 18%) (Table 5). The majority of the cases (*n*=1,109, 72%) were confirmed with laboratory investigations, and patients were primarily treated within the RU (*n*=953, 74%) (Table 5). Treatment at the RU level was high (>75%) for hypertension, diabetes, and asthma and lowest (6%) for cancers. The mean age for all first-time diagnoses was higher in women (45.5 years) than in men (42.5 years) (Table 6). Except for asthma and oral cancers, the mean age for a primary NCD diagnosis varied between 48.9 (hypertension) and 57.4 (CVD) years, respectively, for women and men. Nearly one third of all cases (*n*=345, 29%) were diagnosed in patients younger than 40 years. Logistic regression revealed that the likelihood of diagnosis of metabolic syndrome was higher in patients aged 40–60 years (beta, 2.169; 95% CI, 1.595–2.949) and greater than 60 years (beta, 2.183; 95% CI, 1.470–3.243). Also, with reference to homeopathic practitioners, allopathic practitioners (beta, 1.782; 95% CI, 1.231–2.581) who attained more than 30 patients per day (beta, 1.526; 95% CI, 1.155–2.016) were more likely to diagnose metabolic syndrome cases. Results holds true for diagnosis of cardiovascular disease also. Another model for diagnosis of respiratory diseases revealed that practitioners attending patients below 40 years (beta, 3.520; 95% CI, 2.362–5.246) and 40–60 years (beta, 2.907; 95% CI, 1.713–4.933) with primary school (beta, 1.833; 95% CI, 1.103–3.046) or secondary schooling (beta, 2.546; 95% CI, 1.417–4.576) were more likely to be diagnosed with respiratory diseases. These three diseases diagnosis models were independent of gender of the patients.

**Table 5:** Number of reported cases, confirmation, and treatment pattern

	Hypertension, *n* (%)	Diabetes, *n* (%)	Asthma, *n* (%)	IHD, *n* (%)	COPD, *n* (%)	Cancer, *n* (%)	CVD, *n* (%)	Total (diagnosed cases), *n* (%)	Total (patients), *n* (%)
No. of cases	622 (41)	460 (30)	210 (14)	81 (5)	76 (5)	45 (3)	38 (3)	1,532 (100)	1,283 (100)
Confirmed diagnosis	368 (59)	447 (97)	98 (47)	71 (88)	58 (76)	38 (84)	29 (76)	1,109 (72)	878 (68)
Treatment at facility	485 (78)	357 (78)	184 (88)	40 (49)	48 (63)	4 (6)	12 (32)	1,130 (74)	953 (74)

IHD=ischemic heart disease; COPD=chronic obstructive pulmonary disease; CVD=cardiovascular disease.

Cancer (oral, lung, cervical, and breast) cases have been grouped because of low numbers; two cases with diagnoses missing were excluded from calculation.

**Table 6:** Mean age, gender and education for 10 NCDs

		Hypertension, *n* (%)	Diabetes, *n* (%)	Asthma, *n* (%)	IHD, *n* (%)	COPD, *n* (%)	Cancer, *n* (%)	CVD, *n* (%)	Total (patients), *n*=1,283 (%)
Gender	Men	368 (59)	244 (53)	118 (56)	41 (51)	49 (65)	12 (27)	27 (71)	720 (56)
	Women	245 (39)	210 (46)	91 (43)	37 (46)	24 (32)	33 (73)	9 (24)	542 (42)
Mean age diagnosis	Total	48.1	50.5	34.8	55.6	52.0	53.9	57.4	46.6
	Men	46.7	48.9	33.8	53.9	49.7	46.1	55.0	42.5
	Women	50.2	52.3	36.1	56.5	56.2	56.6	61.3	45.5
Education (age ≥21) (*n*=1,221)	No formal education	72 (12)	67 (16)	29 (20)	12 (17)	23 (32)	5 (14)	10 (29)	162 (13)
	Primary school	110 (19)	84 (20)	26 (18)	15 (21)	15 (21)	8 (22)	1 (3)	214 (18)
	Secondary school	169 (29)	134 (32)	35 (24)	23 (32)	18 (25)	10 (27)	12 (34)	346 (28)
	Graduate degree	176 (30)	119 (28)	49 (34)	16 (23)	12 (17)	11 (30)	9 (26)	337 (28)
	Postgraduate degree	53 (9)	18 (4)	6 (4)	5 (7)	4 (6)	3 (7)	3 (9)	74 (6)

NCD=non-communicable disease; IHD=ischemic heart disease; COPD=chronic obstructive pulmonary disease; CVD=cardiovascular disease.

Missing values for age (*n*=87) and education (*n*=109) not considered in calculation.

In all three areas, majority of patients (*n*=975, 76%) came from the same administrative area (ward) where the respective RUs were located, often even from the same subward (29%, *n*=361) in the two inner city wards. The share of patients without formal educational degree or primary degree was the highest among homeopathic practitioners (*n*=134, 45%), whereas the share of patients with a graduate or postgraduate degree was the highest among allopathic practitioners (*n*=338, 48%).

## Discussion

### Transferability of the study design and key recommendations

The low dropout rate, acceptable respondent cooperation, reporting consistency and data quality, and the positive evaluation of the surveillance study indicate that the inclusion of private practitioners in NCD surveillance is feasible and the study design in principle transferrable to the city of Pune. Findings from this study suggest future NCD surveillance systems should take into account the following challenges.

#### Registration

The selection of RUs for a surveillance system is difficult in urban areas such as Pune due to the lack of a common registration platform for private practitioners. Other issues include lack of continuity, e.g. frequent changes in clinic locations, irregular hours of operation, and periods of absence ((17) for India). A common central registry for all private practitioners irrespective of the system of medicine (on local or national level) with information on medical qualification and specialization – as envisaged by the Clinical Establishments (Registration and Regulation) Act, 2010 (23) – is an important prerequisite to identify RUs for a sentinel surveillance system.

#### System of medicine

In our study, 70% of all NCD cases were diagnosed by ayurvedic and homeopathic practitioners; 70% of these patients were treated at the RU level and not referred. Alternate medicine practitioners are legally only allowed to dispense allopathic drugs in some states in India and also in Maharashtra (24, 25). The state government in Maharashtra allows homeopathic practitioners passing a 1-year course in modern pharmacology to provide allopathic treatment (26). Furthermore, similar to other studies (27, 28), our findings indicate that people of lower socioeconomic status tend to visit alternate medicine practitioners. Excluding them might cause information inequity bias though quality of NCD diagnosis and treatment remains an issue (24, 29).

The inherent problem of distinct diagnostic procedures and nomenclature of diseases within the different systems of medicine (inbuilt barrier) and cross-system practices (24, 30) need to be tackled. Those alternate medicine practitioners providing allopathic treatment for NCDs should be included in NCD surveillance, but their identification may be challenging due to legal constraints.

#### Infrastructure and capacity

The results of the KAP survey and the surveillance study show that the private primary healthcare sector (all systems of medicine) mainly consists of small clinics with limited data-keeping practices (especially lack of electronic medical records), infrastructure (e.g. computer, diagnostic facilities, human resources), and limited availability of time on the part of the practitioner. The data collection tool should therefore be designed in a way that it can be integrated into the clinic's workflow and is simple, easy, and quick to fill in. It should ideally also serve as a benefit for the clinic so as to improve the motivation to fill it.

#### Reporting format and content

Although timeliness of reporting is less critical for NCD surveillance and personal interaction was an important facilitator for setting up and running such a system, onsite data collection is human resource intensive and therefore less practical for a large geographical area on a monthly basis. Although computer availability remains a challenge, paper-based reporting offers limited flexibility for changing content (e.g. to capture upcoming public health issues). The prevailing limited use of electronic medical record system could be used to establish a standardized electronic medical record system in sentinel sites for data reporting based on standardized case definitions that increases data quality (7, 31).

In addition to our reporting form, important NCD risk factors (e.g. body mass index, nutrition, physical activity, stress, family history) and comorbidities with NCDs and communicable diseases should be captured. Important challenges remain the integration of socioeconomic indicators, the absence of a unique patient ID, and the capture of a denominator for the RUs (i.e. total number of patient visits per RU on a daily basis). Furthermore, national standard case definitions for NCDs have to be developed, taking into consideration different systems of medicine in India and the problem of cross-system practice. Given that unique patient identifiers are provided in a system, it would also be useful to capture the treatment process during follow-up visits to provide information on treatment outcomes for NCDs.

#### Knowledge and attitude

The willingness and continuity in participation was higher in ayurvedic and homeopathic practitioners, although allopathic practitioners had a better knowledge about surveillance and submitted a higher number of cases. The surveillance study revealed several behavioral barriers such as lack of interest and commitment in some practitioners. Other practitioners raised the point of difficult relationships with the government sector (i.e. bad experience in cooperation with government sector, e.g. when reporting mandatory cases). Therefore, awareness programs to increase knowledge about disease surveillance and regular interaction and dialogue with participating practitioners are required. Facilitators for continuous reporting of high-quality data are, for example, regular feedback through reports, training and updates on standard case definitions, personal interaction, staff for troubleshooting, and acknowledgment through certificates or credit points.

#### Surveillance approach

Because no comprehensive national framework for regular NCD surveillance currently exists in India, we suggest an augmented sentinel NCD surveillance system on a voluntary basis for documenting and monitoring the ongoing epidemiological changes in urban India. A sentinel system would comply with the requirements to estimate prevalence and incidence rates for specific diseases in different population subgroups and to identify trends (e.g. changes in the disease onset, comorbidities, and treatment outcomes). An NCD surveillance system should include private and public facilities of all healthcare levels. An augmented system with regular contact and feedback would help to increase reporting consistency. Given the complex disease etiology of NCDs, the combination of a sentinel augmented facility-based surveillance with community-based surveys can provide extended information on risk factors, access to treatment, and treatment outcome (7).

## Limitations of the study

We used a paper-based system because this system was the preferred choice by the practitioners, and not all RUs were equipped with computers. It was not possible to collect information on the total number of patients seen by a practitioner on a daily basis; thus, the prevalence per number of patients could not be ascertained. Both of these factors make it difficult to replicate the system design in its current form on a large scale. The evaluation of the implementation process might be biased because only practitioners with a high motivation level participated in the surveillance study.

We used educational qualification as a proxy indicator for the socioeconomic status of the patient, although univariate measures are insufficient to assess socioeconomic status (32). To reduce the amount of time for case recording, data on NCD risk factors and comorbidities were not collected.

With respect to confirmation of the diagnosis, it cannot be ruled out that some practitioners may have recorded cases as ‘confirmed’ instead of ‘presumptive’ diagnosis because of social desirability bias. It can also be assumed that confirmed cases of hypertension are underreported because some practitioners did not consider blood pressure reading as confirmation. We did not provide case definitions due to lack of standardized diagnostic criteria for all conditions and across the different systems of medicine.

## Conclusions

The increasing NCD burden in India and its impact on population health require the implementation of a routine facility-based NCD surveillance system. The findings of the surveillance study indicate that the private primary healthcare sector consisting of allopathic and alternate medicine practitioners is an important source for NCD diagnosis and care and that its involvement in NCD surveillance is possible. Different barriers were identified that have to be addressed, i.e. inbuilt, infrastructural, capacity, knowledge, and behavioral barriers.

Against the current legal background and the heterogeneity of the private healthcare sector in Pune, we suggest an augmented sentinel NCD surveillance system on a voluntary basis among private healthcare facilities and on mandatory basis for public healthcare facilities of all healthcare levels.

## Paper context

Despite the rising impact of NCDs in India, lack of quality data due to missing routine surveillance and disintegration of private healthcare providers hampers NCD prevention and control. Our study tested the inclusion of private primary practitioners into a sentinel NCD surveillance system. Findings indicate that their integration is feasible if major barriers are addressed. We suggest setting up an augmented sentinel surveillance system on selected NCDs on a voluntary basis.
